# Receiving preferred treatments may improve low back pain outcomes: a non-randomized comparative study

**DOI:** 10.3389/fpain.2026.1687883

**Published:** 2026-02-20

**Authors:** Tareq I. Alshyab, Ahmad Alrawashdeh, Nizar Mohammad Audat, Khader A. Almhdawi, Alaa Oteir, Zaid Aldahamsheh, Mohammad A. Bani Hani, Saddam F. Kanaan

**Affiliations:** 1 Department of Rehabilitation Sciences, Jordan University of Science and Technology, Irbid, Jordan; 2 Department of Rehabilitation Sciences, Faculty of Applied Medical Sciences, Jordan University of Science and Technology, Irbid, Jordan; 3 Physical Medicine and Rehabilitation, Jordanian Royal Medical Services, Amman, Jordan; 4 College of Health Sciences, QU Health, Qatar University, Doha, Qatar

**Keywords:** disability, low back pain, mental health, pain, patient preference, patient-centered care, physical therapy, rehabilitation

## Abstract

**Purpose:**

The aim of this study was to compare health-related outcomes between individuals with low back pain (LBP) who received their preferred treatments and those who received non-preferred treatments.

**Methods:**

A non-randomized, multicenter, comparative study was conducted. Patients with chronic LBP (duration >3 months) were recruited from military hospitals in Jordan. The LBP Treatment Beliefs Questionnaire (TBQ) was used to determine participants’ treatment preferences across five treatment options. Baseline assessments of all outcome measures were compared with post-treatment assessments using multivariate and univariate analyses of variance.

**Results:**

A total of 205 participants completed the study with a mean age of 42.6 (±12.9). According to TBQ, 131 participants (63.9%) received their preferred treatment. Compared with the non-preferred group, the preferred group demonstrated a statistically significant (*p* < 0.001) decrease in numeric pain rating scores (adjusted mean difference = −0.87), Oswestry Disability Index scores (adjusted mean difference = −4.64), Depression Anxiety Stress Scale (DASS)-stress (adjusted mean difference = −1.91), DASS-anxiety (adjusted mean difference = −2.11), and DASS-depression (adjusted mean difference = −1.94).

**Conclusion:**

Incorporating patients' treatment preferences into treatment plans may positively influence pain, functional outcomes, and psychological wellbeing.

## Introduction

Despite recent advances in LBP diagnosis and treatment, the disease burden on patients and healthcare systems continues to steadily increase (1). In 2021, approximately 628.8 million people worldwide were affected by LBP, making it the most prevalent musculoskeletal disorder in terms of age-standardized disability-adjusted life years (2). LBP not only affects physical function but also exerts a substantial psychosocial toll on individuals, underscoring the need for more comprehensive treatment approaches (3, 4).

In addition to guidelines, evidence-based practice, and clinical reasoning, clinicians are increasingly incorporating patients' treatment preferences into clinical decision-making regarding optimal LBP treatment (5–7). However, few studies have examined the potential benefits of incorporating patients' treatment preferences into LBP treatment outcomes.

Treatment preference refers to a patient's choice among available treatment options for a clinical condition (8). Understanding patients' expectations and treatment preferences can guide treatment selection and, in some cases, improve LBP treatment outcomes (9, 10). Current evidence-based LBP guidelines encourage healthcare practitioners to balance the best available evidence with patient preferences when developing a treatment plan (11). Therefore, clinicians are urged to actively engage patients in selecting interventions by providing balanced, evidence-based information, clarifying the options and potential outcomes, supporting deliberation, and ensuring that treatment decisions reflect patients' values and preferences (12).

Clinicians typically select treatment programs based on established guidelines, evidence-based practices, and/or clinical reasoning. Patients' treatment preferences may be informed by prior treatment experiences, knowledge, beliefs, attitudes toward LBP, reading, social media, or advice from experts and friends (13–15). These preferences can influence treatment engagement, adherence, psychological responses, and ultimately treatment clinical outcomes (13, 16).

A randomized clinical trial by Bishop et al. demonstrated that treatment preferences can positively influence pain outcomes in individuals with LBP (17). In that study, 60 participants were randomly assigned to receive one of two manual therapy approaches (joint-biased or constant touch). However, the trial was limited by its small sample size and narrow focus, as it did not explore the full range of available treatment options or evaluate outcomes beyond pain. These limitations highlight the need for broader investigations into how incorporating patient treatment preferences into clinical decision-making might influence multiple domains of LBP outcomes, including disability, psychological wellbeing, and fear-avoidant beliefs (9).

While emerging evidence suggests the benefits of preference-based treatment approaches, comprehensive studies remain necessary to fully understand the potential benefits on LBP treatment outcomes. Therefore, the present study aimed to investigate the effect of receiving preferred treatments on LBP outcomes compared to receiving non-preferred treatment among individuals with LBP. We hypothesized that patients who received treatment consistent with their preference would demonstrate less pain intensity, reduced disability, fewer mental health symptoms, and lower fear avoidance scores than the participants who did not receive their preferred treatment. The findings from this study may inform clinical decision-making regarding the most appropriate treatment to optimize LBP management.

## Materials and methods

### Study design and settings

A multicenter, prospective, non-randomized comparative study was conducted at Princess Haya Bient Al Hussein and Prince Rashed Bin Al-Hasan Military Hospital in Jordan between February and June 2021.

### Sample and recruitment

Eligible participants were patients older than 18 years with chronic LBP (>3 months), reporting an average pain score of more than 3/10 over the previous 8 weeks, and receiving physical therapy services for the first time (so that their previous experiences do not affect the choices of treatments). Exclusion criteria included patients diagnosed with cancer, ankylosing spondylitis, or inflammatory arthritis; patients with postoperative or post-traumatic LBP; and patients with LBP due to pregnancy or childbirth. We also excluded patients with mental disorders, neurological diseases, or cognitive or communication deficits that prevented them from communicating with the researchers and therapists. All patients matching the inclusion criteria were invited to participate in the study and provided consent. The Jordan University of Science and Technology Institutional Review Board approved the study (#52/137/2021).

### Treatment

Participants were assigned to a physical therapist from the departmental staff. In this pragmatic clinical setting, allocation to a physical therapist followed the hospital's routine scheduling procedures (based on therapist availability and appointment slots) rather than patient choice or clinical profile, and there was no attempt to allocate patients according to their treatment preferences. As part of standard care in the physical therapy unit at Royal Medical Services, each patient received six physical therapy sessions (two sessions per week for 3 weeks). Each session lasted 30–60 min. Physical therapists provided individualized treatment based on physical therapy evaluations. All physical therapists who provided treatment to study participants were blinded to the study's aim and patients’ treatment preference classification. We documented whether the referring physician had prescribed pain medications. In addition, we cross-checked whether the patients were using these medications. The treatment provided by physical therapists was documented for each patient and compared with the patients' preferred treatments. Participants were assessed at baseline and after completing the treatment program (six sessions in 3 weeks). Participants were blinded to the study aim and hypothesis; however, outcome assessors were not blinded because outcome measures were collected via self-reported questionnaires completed directly by participants.

### Data collection

An independent therapist assessed patients using the following measures:

1- Sociodemographic characteristics were collected at baseline, including age, gender, body mass index (BMI), education, occupation, number of children, and residential areas (urban vs. suburban).
2- Clinical and lifestyle characteristics were noted, including duration of low back pain, consumption of painkillers, comorbidities, smoking habits, and daily hours of sports activity.
3- Treatment preference was measured at baseline using the LBP Treatment Beliefs Questionnaire (LBP-TBQ). LBP-TBQ systematically collects patients' beliefs about several therapeutic procedures for LBP (14). The Arabic-translated questionnaire consists of eight Likert scale items with three subscales—“credibility (items 1 and 2),” “effectiveness and fit (items 3, 4, and 5),” and “concerns (items 6, 7, and 8)”—for each of the following common LBP interventions: pain medication, exercise, manual therapy, electro-heat therapy, and surgery (18).
  According to the LBP-TBQ results, treatment preference was classified as highly preferred (score = 4), moderately preferred (score = 3), minimally preferred (score = 2), least preferred (score = 1), or not preferred (score = 0). The average score was calculated by summing the provided treatment scores and dividing by the number of treatments provided. Treatment was considered consistent with a patient's preference (preferred) if the average TBQ score was ≥2, and inconsistent (non-preferred) if the average score was <2. Because no validated cutoff exists in prior literature, the threshold was determined through consensus among the research team after reviewing the scale structure and distribution of the pilot data. Although surgery was not a treatment option in the present study, it was included in the LBP-TBQ to capture the full range of patients' treatment beliefs. Participants who indicated a strong preference for surgery typically demonstrated lower preference scores for conservative interventions and were therefore more likely to be classified as not receiving their preferred treatment.
4- The Numeric Pain Rating Scale (NPRS) (19) was used to measure the pain intensity. The NPRS is an 11-point Likert scale administered verbally, ranging from 0 (no pain) to 10 (worst pain imaginable). The difference of at least two points on the NPRS scale is clinically important (20).
5- The Oswestry Disability Index (ODI) was used to measure disability resulting from LBP (21). It is designed to assess the limitations of various activities in daily life. The total score ranges from 0% to 100%, with higher scores indicating greater disability (22). A change of 12.72% in ODI is considered clinically meaningful (23).
6- The Depression Anxiety Stress Scale (DASS-21) was used to measure the emotional state of depression, anxiety, and stress. DASS-21 includes three subscales to measure the emotional states of depression, anxiety, and stress. The cutoff points are reported to be 9 for depression, 7 for anxiety, and 14 for stress, with higher scores indicating mild to extremely severe symptoms (24).

The Fear Avoidance Beliefs Questionnaire (FABQ) was used to assess the impact of patients' fear-avoidant beliefs regarding physical activity and work on LBP disability. Higher FABQ scores indicate stronger fear (25). A total change of 11 points is considered clinically meaningful (26).

### Statistical analysis

An *a priori* sample size estimation was performed using G*Power 3.1 (27) based on pilot data (*n* = 10) from change scores of the proposed outcome measures, which yielded effect sizes ranging from 0.57 to 1.01 (Cohen's *d*). To ensure adequate statistical power, the calculation was based on the smallest observed effect size (*d* = 0.57) for an independent-samples *t*-test (two-tailed, *α* = 0.05, power = 0.80). Adjustment for multiple comparisons was not applied, because the multivariate analysis of covariance (MANCOVA) global test was considered the primary analysis. This indicated a minimum of 49 participants per group. This approach was selected to provide sufficient power for both the planned MANCOVA and the subsequent outcome-specific analysis of covariance (ANCOVA) models. Fewer than 5% of data were missing across outcome measures, and missing values were handled using multiple imputation by chained equations, consistent with the recommended practice for low-level missing data (28). To account for potential dropouts and increase precision, we recruited at least 74 participants per group.

As appropriate, sociodemographic data were summarized as means and standard deviations (SDs) or frequencies and proportions (%). The received treatment was dichotomized based on its consistency with the preferred treatment [preferred (TBQ ≤ 2) or non-preferred (TBQ < 2)]. Change scores for all treatment outcome measures were calculated by subtracting baseline scores from post-treatment scores. To account for the non-randomized design and the related significant difference between preferred and non-preferred groups in baseline ODI (*p* = 0.014), a general linear model using the MANOVA test was conducted to investigate differences in change scores between groups (preferred vs. non-preferred), including all outcome measures in the model. When the overall multivariate test showed a significant group effect on change scores, we conducted separate ANCOVA models for each outcome to compare change scores between the preferred and non-preferred groups, with group as the fixed factor and baseline ODI as a covariate. We estimated adjusted between-group differences, 95% confidence intervals, and effect sizes from the ANCOVA models while controlling for baseline ODI. We used SPSS (Statistical Package for the Social Sciences) version 31 (SPSS Inc., Chicago, IL, USA) for all statistical analyses.

## Results

Due to the pragmatic nature of the study and the use of routine clinical recruitment, the total number of individuals approached or screened for eligibility was not systematically recorded. A total of 205 participants who met the inclusion criteria and provided informed consent were enrolled in the study. Complete baseline and post-treatment data were available for all enrolled participants. At baseline, 131 participants (63.9%) later received their preferred treatment, whereas 74 (36.1%) did not. Table 1 summarizes the demographic and clinical characteristics of the preferred and non-preferred groups. No baseline differences were detected for age, BMI, pain intensity, psychological measures, or fear-avoidant beliefs. However, the non-preferred group reported a significantly higher ODI than the preferred group (57.1 vs. 52.2; *p* = 0.014).

**Table 1:** Participants’ demographics and baseline clinical characteristics.

Variable	Total 205 (100%)	Preferred 131 (63.9%)	Non-preferred 74 (36.1%)	*p*-Value
Age in years, mean (SD)	42.6 (12.9)	42.9 (12.7)	42.1 (13.4)	0.676^a^
BMI, mean (SD)	27.7 (4.7)	27.8 (4.6)	27.4 (5.0)	0.601^a^
Children, mean (SD)	3.7 (3.0)	3.8 (3.0)	3.6 (3.1)	0.798^a^
Gender				
Male	100 (48.8)	63 (48.1)	37 (50.0)	0.884^b^
Female	105 (51.2)	68 (51.9)	37 (50.0)	
Marital state				
Single, divorced, widowed	32 (15.6)	23 (17.6)	9 (12.2)	0.423^b^
Married	173 (84.4)	108 (82.4)	65 (87.8)	
Smoking				
Cigarettes	72 (35.1)	41 (31.3)	31 (41.9)	0.266^b^
Water pipe	37 (18.0)	24 (18.3)	13 (17.6)	
No smoking	96 (46.8)	66 (50.4)	30 (40.6)	
Highest education level				
High school	123 (60.0)	75 (57.3)	48 (64.9)	0.349^b^
Diploma	38 (18.5)	28 (21.4)	10 (13.5)	
Bachelor or higher	44 (21.5)	28 (21.4)	16 (21.6)	
Work				
Work	107 (52.2)	68 (51.9)	39 (52.7)	0.913^b^
Not work	98 (47.8)	63 (48.1)	35 (47.3)	
Pain killer				
Yes	138 (67.3)	85 (64.9)	53 (71.6)	0.355^b^
No	67 (32.7)	46 (35.1)	21 (28.4)	
Comorbidities				
Yes	71 (34.6)	47 (35.9)	24 (32.4)	0.649^b^
No	134 (65.4)	84 (64.1)	50 (67.7)	
Sports hours (per week)				
Less than 1 h	162 (79.0)	104 (79.4)	58 (78.4)	0.698^b^
1–4 h	25 (12.2)	17 (13.0)	8 (10.8)	
More than 4 h	18 (8.8)	10 (7.6)	8 (10.8)	
Clinical characteristics, mean (SD)				
Low back pain duration (months)	19.9 (17.0)	20.1 (15.2)	19.6 (19.8)	0.851^a^
Numeric pain rating scale	7.6 (1.3)	7.6 (1.4)	7.8 (1.1)	0.331^a^
Oswestry disability index	53.9 (13.9)	52.2 (14.7)	57.1 (11.9)	0.014^a^
DASS-stress	10.6 (4.0)	10.3 (5.3)	11.2 (4.5)	0.225^a^
DASS-anxiety	6.8 (4.4)	7.0 (4.9)	6.4 (4.3)	0.346^a^
DASS-depression	6.8 (4.4)	7.1 (4.8)	6.3 (3.5)	0.266^a^
FABQ-total score	42.0 (9.7)	41.7 (10.2)	42.3 (9.3)	0.704^a^

All results are presented as *n* (%) unless otherwise indicated.

BMI, body mass index; DASS, depression anxiety stress scales; FABQ, fear avoidance beliefs questionnaire.

a Independent sample *t*-test.

b Chi-square test.

As shown in Table 2, significant reductions were observed postintervention in NPRS (mean difference = −3.07, 95% CI −2.84 to −3.31; *p* < 0.001), ODI (−16.85, 95% −14.98 to −18.73; *p* < 0.001), DASS-stress (−3.78, −3.25 to −4.31; *p* < 0.001), DASS-anxiety (−2.48, 95% CI −2.93 to −2.04; *p* < 0.001), DASS-depression (−2.52, 95% CI −2.09 to −2.96; *p* < 0.001), and FABQ (−12.93, −10.46 to −15.41; *p* < 0.001), indicating notable improvements following six physiotherapy sessions.

**Table 2:** Paired sample *t*-test between the changes from baseline to the end of 3 weeks of physiotherapy (six sessions).

Outcome measures	Baseline	Three weeks	Mean difference	95% CI	T	*p*-Value
Numeric pain rating scale	7.64 (1.30)	4.57 (1.97)	−3.07	−3.31 to −2.83	25.83	<0.001
Oswestry disability index	53.94 (13.91)	37.09 (14.40)	−16.85	−18.72 to −14.98	17.73	<0.001
Depression anxiety stress scale						
Stress	10.59 (4.98)	6.81 (4.49)	−3.78	−4.31 to −3.25	14.04	<0.001
Anxiety	6.76 (4.66)	4.28 (4.03)	−2.48	−2.93 to −2.04	11.00	<0.001
Depression	6.79 (4.36)	4.27 (3.97)	−2.52	−2.96 to −2.08	11.38	<0.001
Fear avoidance belief questionnaire total score	42 (9.67)	29.07 (12.33)	−12.93	−15.38 to −10.48	10.41	<0.001

The overall MANCOVA revealed a statistically significant difference in change scores across all outcome measures between the preferred and non-preferred groups [Wilks' Lambda = 0.902, *F* (5, 199) = 4.29, *p* < 0.001]. Consequently, we conducted a separate ANCOVA for each outcome measure. Table 3 presents the adjusted between-group differences in change scores for outcome measures (from baseline to the end of treatment). After adjustment, the reduction in NPRS scores was significantly greater in the preferred group compared to the non-preferred group (adjusted mean difference = −0.87, 95% CI: −1.35 to −0.39; *p* > 0.001). Although statistically significant, this change did not reach the clinically meaningful threshold of two points.

**Table 3:** Adjusted between-group differences in outcome change scores (ANCOVA adjusted for baseline ODI).

Outcome measures	Change score		Adjusted mean difference	95% CI	*t*	*p*-Value	Cohen's *d*
	Preferred adjusted mean (SE)	Non-preferred adjusted mean (SE)					
Numeric pain rating scale	−3.74 (0.22)	−2.87 (0.24)	−0.87	−1.35 to – 0.39	−3.61	<0.001	−0.51
Oswestry disability index	−22.61 (1.62)	−17.97 (1.71)	−4.64	−8.48 to −0.80	−2.38	0.018	−0.34
DASS-stress	−5.92 (0.39)	−4.01 (0.41)	−1.91	−2.99 to −0.82	−3.46	0.001	−0.49
DASS-anxiety	−11.68 (0.62)	−9.57 (0.66)	−2.11	−3.39 to −0.83	−3.26	0.001	−0.46
DASS-depression	−3.41 (0.27)	−1.47 (0.29)	−1.94	−2.89 to −0.99	−4.02	<0.001	−0.57
Fear avoidance belief questionnaire total score	−14.83 (1.05)	−11.62 (1.11)	−3.21	−6.77 to 0.34	−1.79	0.076	−0.25

Values represent estimated marginal means (± SE) derived from ANCOVA models adjusted for baseline ODI.

The decrease in ODI was significantly greater in the preferred group (adjusted mean difference = −4.64; 95% CI: −8.48 to −0.80; *p* = 0.018). This difference is less than the clinically meaningful change of 12.72.

For emotional symptoms, the preferred group showed significantly larger reductions in DASS-stress (adjusted mean difference −1.91, 95% CI: −2.99 to −0.82; *p* = 0.001), DASS-anxiety (adjusted mean difference = −2.11; 95% CI: −3.39 to −0.83; *p* = 0.001), and DASS-depression (adjusted mean difference −1.94, 95% CI: −2.89 to −0.99; *p* < 0.001). Although all changes were statistically significant, no clinically meaningful thresholds were defined for the DASS-21 in the current study.

Finally, the adjusted between-groups difference in FABQ was not statistically significant after adjustment for baseline ODI (adjusted mean difference = −3.21, 95% CI: −6.77–0.34; *p* = 0.076).

## Discussion

In this study, we examined the role of patient treatment preference in individuals with LBP and evaluated how receiving a preferred treatment influenced clinical outcomes. Our findings indicate that participants whose treatment aligned with their stated preference demonstrated significantly greater improvements across multiple domains—including pain intensity, functional disability, and emotional wellbeing—compared with those who received a non-preferred treatment.

However, the difference in pain was modest and below the threshold for interpreting individual-level clinically meaningful change. Similarly, the changes in ODI can be interpreted similarly, although concordance with treatment preference remained a significant difference even after adjustment for baseline ODI. This is a very important distinction, indicating that receiving a preferred treatment was associated with incremental benefit rather than overall clinical improvement during the physiotherapy session in both groups. Although changes in emotional symptoms are statistically significant, there are no established clinically meaningful thresholds. However, the magnitude of change in DASS-21 in our study is comparable to that reported in studies of cognitive behavioral therapy and physical therapy for patients with LBP, demonstrating the clinical relevance of our results (29). In contrast, the FABQ did not show a significant difference between groups, which may indicate that fear avoidance is related to factors other than preference.

The results from this study support guideline recommendations that healthcare practitioners balance the best available evidence with patients' preferences when developing a treatment plan (11, 30). In addition, these results confirm the previous findings from Bishop et al.'s study, who reported that participants receiving a preferred treatment experienced improved pain outcomes (17). Importantly, our study extends this body of evidence by employing a comprehensive assessment battery—including pain, disability, emotional status, and fear avoidance—and assessing outcomes in a real-world physiotherapy clinical setting. This approach yields more ecologically valid insights than prior experimental models, thereby reinforcing the applicability of preference-based care in routine practice.

Our results are consistent with the findings of the Patients' Preferences Collaborative Review Group (2008), which analyzed eight randomized musculoskeletal trials and found that participants receiving their preferred treatment tended to have better outcomes than those who did not, although the pooled difference did not reach statistical significance (31). Similar conclusions were reported by Delevry and Le (32) in their systematic review and meta-analysis of 27 clinical trials, which reported that allowing patients to choose their preferred treatment was associated with better clinical outcomes in mental health and pain, with a small but statistically significant pooled effect size of 0.18 (32). Our findings parallel earlier results, showing that in patients with LBP, receiving preferred treatment was associated with greater improvements in pain, function, and mental health symptoms than receiving a non-preferred treatment (31, 32). Recent evidence by Zhu et al. further highlights that in chronic musculoskeletal pain using discrete choice experiments, including LBP, patient treatment preferences cluster around outcomes such as efficacy in pain reduction, functional improvement, risk of adverse events, cost, and convenience, reinforcing how these preferences shape engagement and satisfaction with treatment (33). In addition, Bailly et al., in their clinical guidelines and care pathway for the management of LBP, emphasized that treatment plans should be individualized and explicitly account for patient expectations and preferences, noting that such alignment may enhance adherence, patient satisfaction, and overall effectiveness of care (34).

Beyond musculoskeletal conditions, patient treatment preferences have been shown to influence outcomes in serious or chronic diseases. For example, studies in cancer care demonstrate that honoring patient autonomy and preference, especially regarding end-of-life treatments, can reduce decisional conflict and increase satisfaction (35). According to one study, patients with diabetes whose treatment preferences were considered in treatment decision-making reported greater adherence to the treatment plan and better glycemic control (36). In addition, a landmark randomized controlled trial in poorly controlled asthma demonstrated that patients who participated in shared decision-making—in which treatment plans were developed collaboratively with clinicians based on patient goals and preferences—showed significantly better medication adherence and clinical outcomes after 1 year than those receiving standard care (37).

Many authors have reported that patients who obtain their preferred treatment may be more motivated, adhere more closely to treatment regimens, and report better outcomes. A newly published study shows that Canadian military veterans with chronic LBP support this relationship, showing that patients are more willing to accept treatments that match their preferences, particularly when treatments demonstrate effectiveness, are provided by a trustworthy provider, and are accessible (38). Patients who did not receive their preferred treatment may become resentful, lose motivation, fail to adhere to the treatment program, fail to report accurately at follow-up, or even drop out of the experiment (39). One meta-analysis summarized results from 35 studies that examined the preference effect on treatment adherence and treatment outcomes among adult patients with LBP. Participants were included from different populations and received different treatments. The results showed that participants matched to their preferred therapy showed significantly lower dropout rates (odds ratio = 0.59, *p* < 0.001) and achieved more substantial improvements in treatment outcomes (effect size *d* = 0.31, *p* < 0.001) (40). Thus, according to our study results, treatment preference may play an essential role in increasing treatment adherence and, consequently, treatment outcomes.

Expectancy and contextual factors can also account for the effects of observed preferred treatment, alongside behavioral and motivational explanations. A sense of alignment between one's personal beliefs and the treatment received is likely to be reinforced when one receives the treatment of their choice. Through well-established neurobiological mechanisms, these expectancy effects can profoundly influence symptom perception and treatment response (41). Stronger positive priors may bias perceptual inference toward less pain and better wellbeing when a patient receives a preferred treatment, according to modern neurocognitive models such as the Bayesian brain framework, which contend that symptom experience results from the brain's integration of prior expectations with incoming sensory information (42). Moreover, contextual factors such as communication style, therapeutic environment, perception of safe and effective care, and perceived effectiveness are increasingly recognized as active components of care that interact with expectations to influence outcomes (43). Although all treatments reflected standard physiotherapy care, participants in our preferred treatment group showed greater improvements across multiple domains, which may be partially explained by the combined pathways of expectancy confirmation and contextual support.

Understanding patients' preferences could have therapeutic benefits by supporting patients' autonomy and enhancing satisfaction with care. Our results support the shift toward patient-centered care in healthcare. Our results encourage clinicians to systematically integrate treatment preferences with evidence-based practice and clinical expertise when developing individualized treatment plans, as recommended by current clinical practice guidelines (44). For example, in their randomized controlled trial (RCT), Suarez-Almazor et al. compared the efficacy of traditional Chinese acupuncture and sham acupuncture and also examined the effects of acupuncturists' communication style in patients with knee osteoarthritis. They found that patients who had a stronger belief that acupuncture might help with osteoarthritis knee pain were more satisfied during treatment, and this satisfaction was associated with better pain outcomes several weeks later (45).

In recent decades, the percentage of patients who prefer to be involved in decision-making with their healthcare professional has risen, along with a greater emphasis on eliciting patients' preferences across healthcare settings (46). Thus, the findings of this study may aid physiotherapists in better understanding how patients with LBP perceive various treatment modalities and consider their preferences when developing a treatment plan.

## Limitations

The study has several limitations. First, given the non-randomized, pragmatic design of the study, some selection or expectation bias may persist. While the groups were generally similar at baseline, unmeasured variables, such as patients' expectations or subtle therapist influences, might have affected both the likelihood of receiving a preferred treatment and their outcomes. Thus, these results reflect an association rather than a certain causal effect. The follow-up period was limited to 6 weeks; a more extended follow-up period, at least 3 months, is needed to confirm our results and explore the effects on long-term outcomes. Second, we included patients with LBP who have had pain for more than 3 months (chronic LBP). Therefore, while our study findings can be generalized to similar groups, caution is warranted when applying them to patients with acute or subacute LBP. Third, we included the patient's first physical therapy visits. Patients' prior experiences may influence treatment preferences for LBP. Moreover, we did not measure patients' adherence, as their treatment preferences may affect adherence. Finally, our study lacked randomization. Therefore, future research should include younger patients and RCTs to assess the effect of patients' treatment preferences on outcomes in patients with LBP.

## Conclusion

Our study demonstrated that receiving preferred LBP treatment was associated with a significantly greater reduction in pain, disability, and mental health symptoms among LBP patients who received their preferred treatment compared to those who did not. Thus, physical therapists are encouraged to consider patients' treatment preferences when developing treatment plans for patients with LBP. Incorporating patients' preferences into treatment decisions may improve outcomes in patients with LBP.

## Data Availability

The raw data supporting the conclusions of this article will be made available by the authors without undue reservation.
